# A Case Report on Solitary Extramedullary Plasmacytoma of the Pleura

**DOI:** 10.7759/cureus.28483

**Published:** 2022-08-27

**Authors:** Shobha Mandal, Saswat Jha, Mary Grace Bethala, Naaila Ali, Rahul Gosain

**Affiliations:** 1 Internal Medicine, Guthrie Robert Packer Hospital, Sayre, USA; 2 Internal Medicine, Kathmandu Medical College and Teaching Hospital, Kathmandu, NPL; 3 Internal Medicine, GlobeHealer, Philadelphia, USA; 4 Medicine, Lake Erie College of Osteopathic Medicine (LECOM), Erie, USA; 5 Hematology and Oncology, Guthrie Corning Cancer Center, Guthrie Robert Packer Hospital, Sayre, USA

**Keywords:** solitary plasmacytoma, solitary extramedullary plasmacytoma, solitary bone plasmacytoma, pleura, plasmacytoma, extramedullary plasmacytoma

## Abstract

Solitary plasmacytoma is an extremely rare form of plasma cell malignancy that presents as a single mass of monoclonal plasma cells located either intraosseous or extramedullary (extraosseous). Extramedullary plasmacytoma can affect any part of the body, but the most common sites of origin are the head and neck region. The involvement of pleura is very rare. Here, we are enlightening this rare presentation and making readers aware of the clinical presentation and management of this rare malignancy.

## Introduction

Plasma cell (PC) neoplasm can present in different forms and can be classified based on the location of the neoplasm. Solitary plasmacytoma (SP) is characterized by the neoplastic proliferation of a single clone of plasma with no or minimal bone marrow (BM) plasmacytosis and with no other symptoms other than those derived from the primary lesion. Based on the pattern of involvement of the bone or soft tissue it is further classified as solitary bone plasmacytoma (SBP) or solitary extramedullary plasmacytoma (SEMP). SBP infiltrates a single bone whereas SEMP infiltrates the soft tissue [1]. SP is an extremely rare malignancy with an incidence of 0.15/100.000 [2]. Among all the cases of SP, 70 % of cases are SBP primarily involving the red marrow-containing bones such as vertebrae, femurs, pelvis, and ribs whereas SEMP involves any other organs except the bone [3]. Even though patients with SEMP and SBP have minimal bone marrow involvement they have a higher risk of developing symptomatic multiple myeloma (MM). After the initial diagnosis, 50% of patients with SBP and 30% of patients with EMP (extramedullary plasmacytoma) were found to progress to MM within 10 years. All patients diagnosed with SP need appropriate treatment and close surveillance by the oncologist [4].

## Case presentation

**Figure 1:**
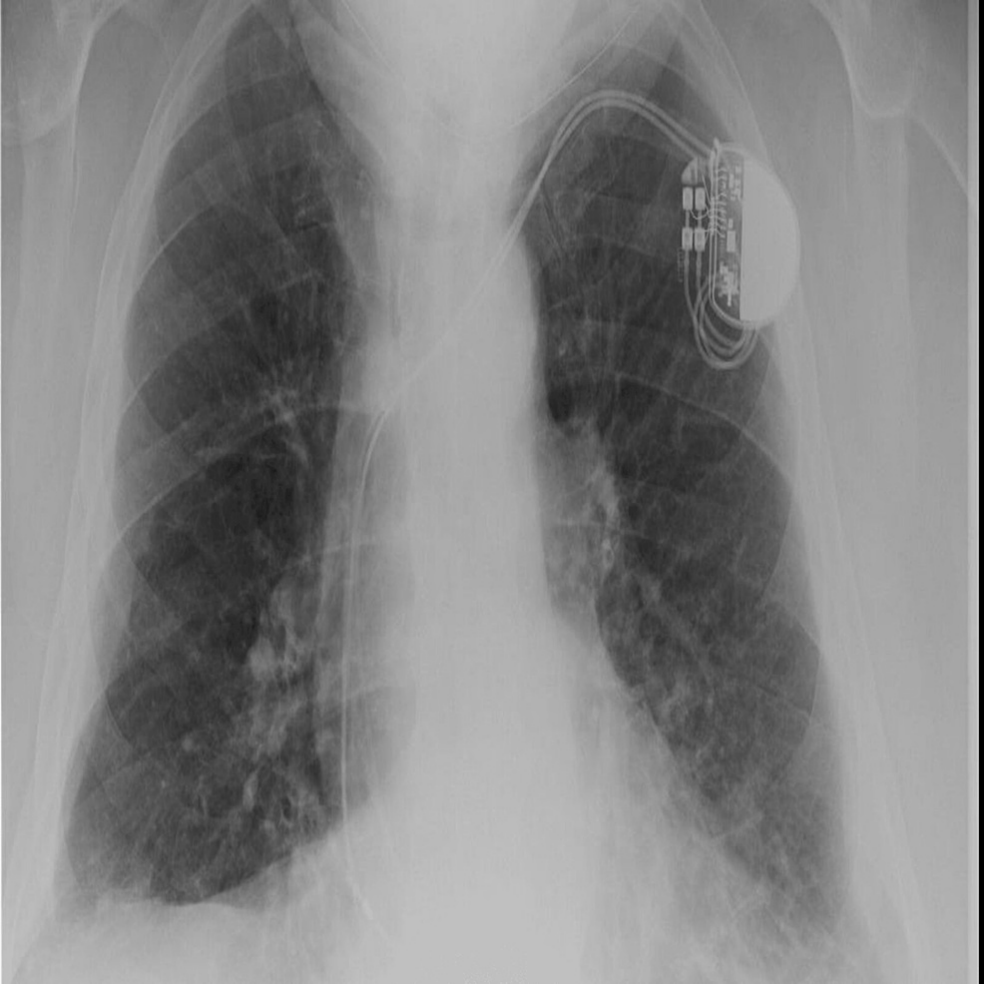
Chest X-Ray showing finding suggestive of left pleural effusion

An 86-year-old gentleman with a past medical history of dyslipidemia, hypertension, and congestive heart failure presented to the primary care provider with a complaint of new onset of bilateral lower extremity swelling and shortness of breath on exertion since last three months. His symptoms were progressively getting worse. On occupational history, he had a long history of exposure to different occupational hazards including asbestos working with pipes. He was a non-smoker. On chest auscultation, he had bilateral basal crackles. He was admitted to the hospital for further workup where he was found to have a left pleural effusion on the chest X-ray (Figure 1).

**Figure 2:**
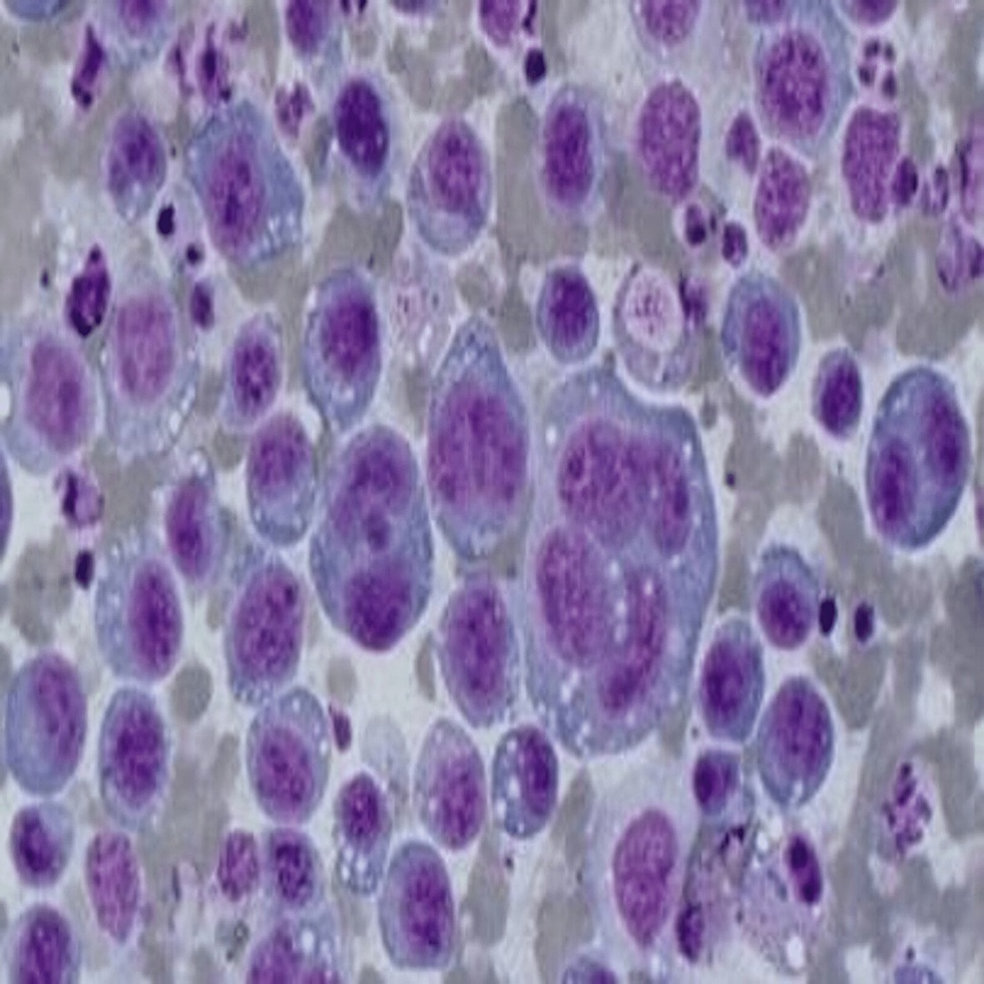
Pleural fluid cytology image Pleural fluid cytology shows atypical plasma cells with binucleation and multinucleation (X 400)

**Figure 3:**
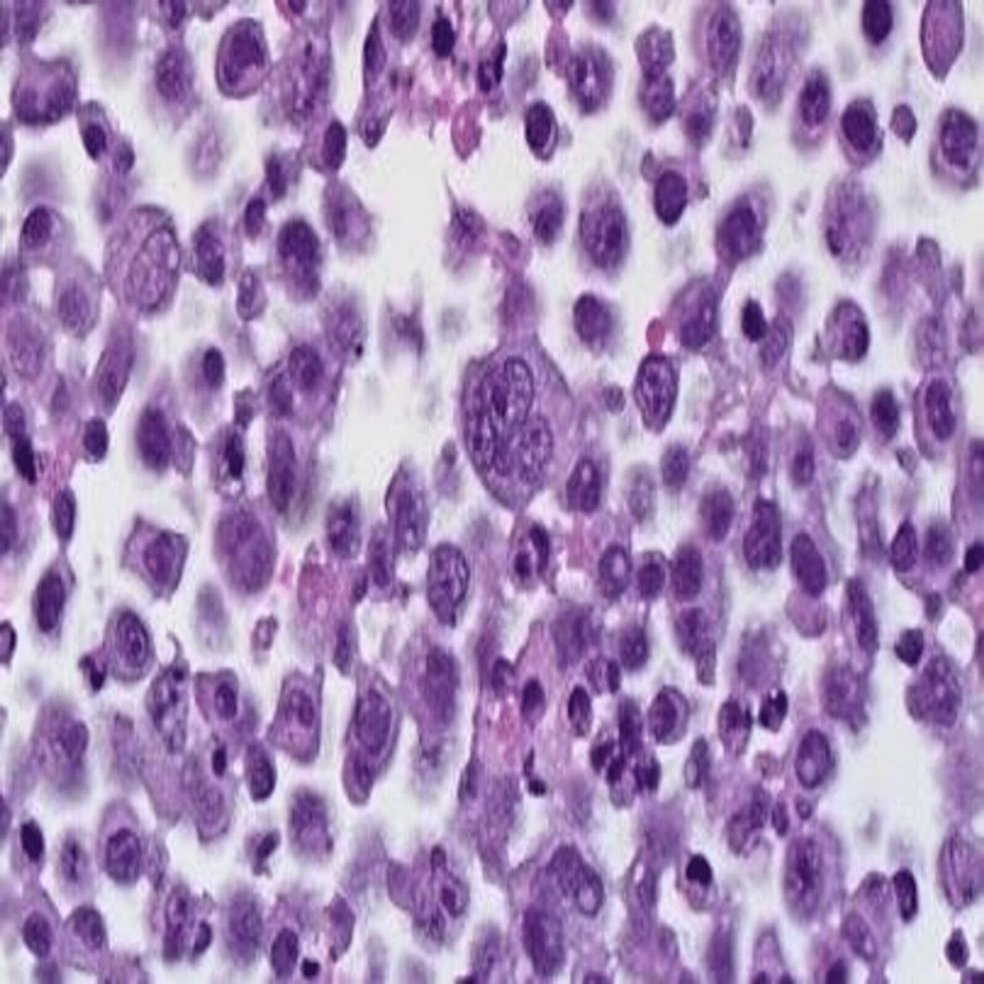
Atypical plasma cells with binucleation and multinucleation (X 400)

On workup, laboratory data showed normal calcium (10.1 mg/dL), average blood urea nitrogen, and creatinine (18 mg/dL and 0.75 mg/dL, respectively), and no evidence of anemia. The patient underwent diagnostic and therapeutic thoracentesis; the pleural fluid cytology was positive for atypical plasmacytoid cells, positive for CD138 (a tumor marker) with aberrant expression of CD10 56 and lambda light chain restriction, and few plasma cells were positive for kappa light chain suggestive of involvement by plasma neoplasm or primary effusion lymphoma (Figures 2, 3). 

**Figure 4:**
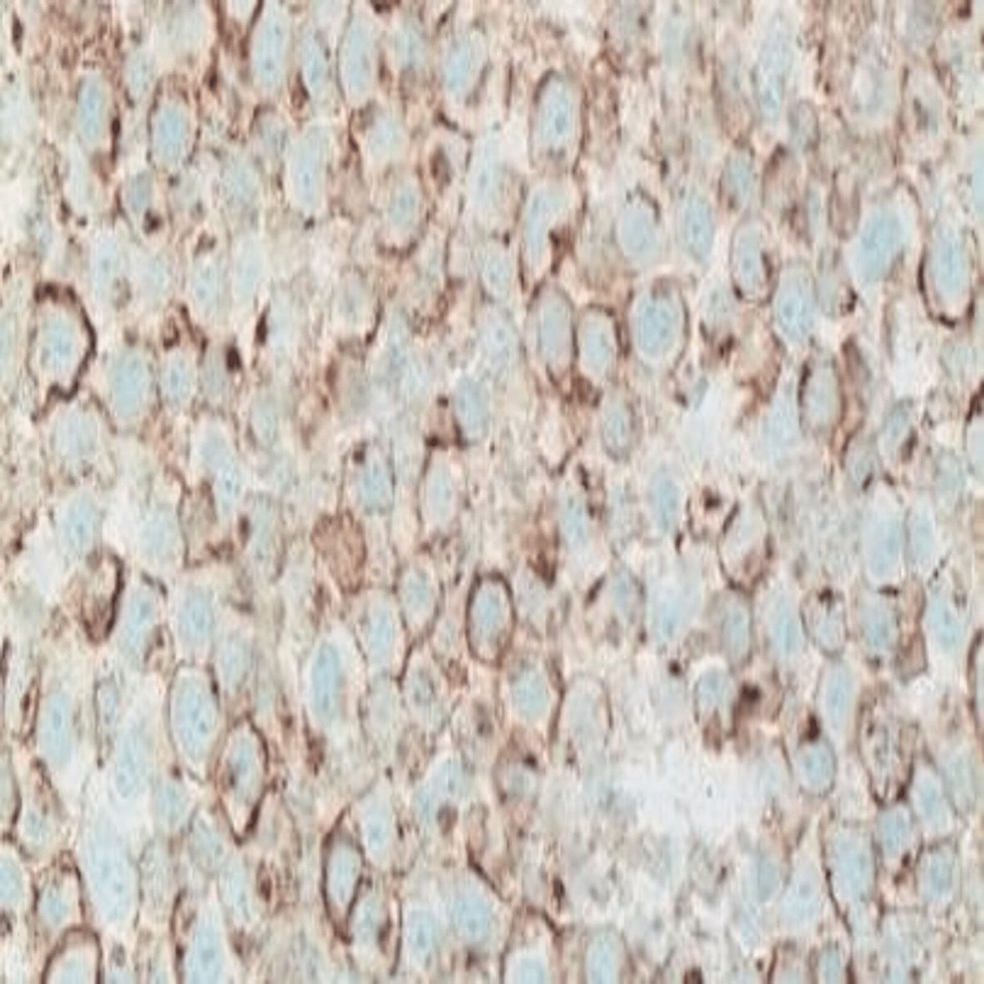
Immunohistochemistry image 1 (X 400) Plasma cells are positive for CD138.

**Figure 5:**
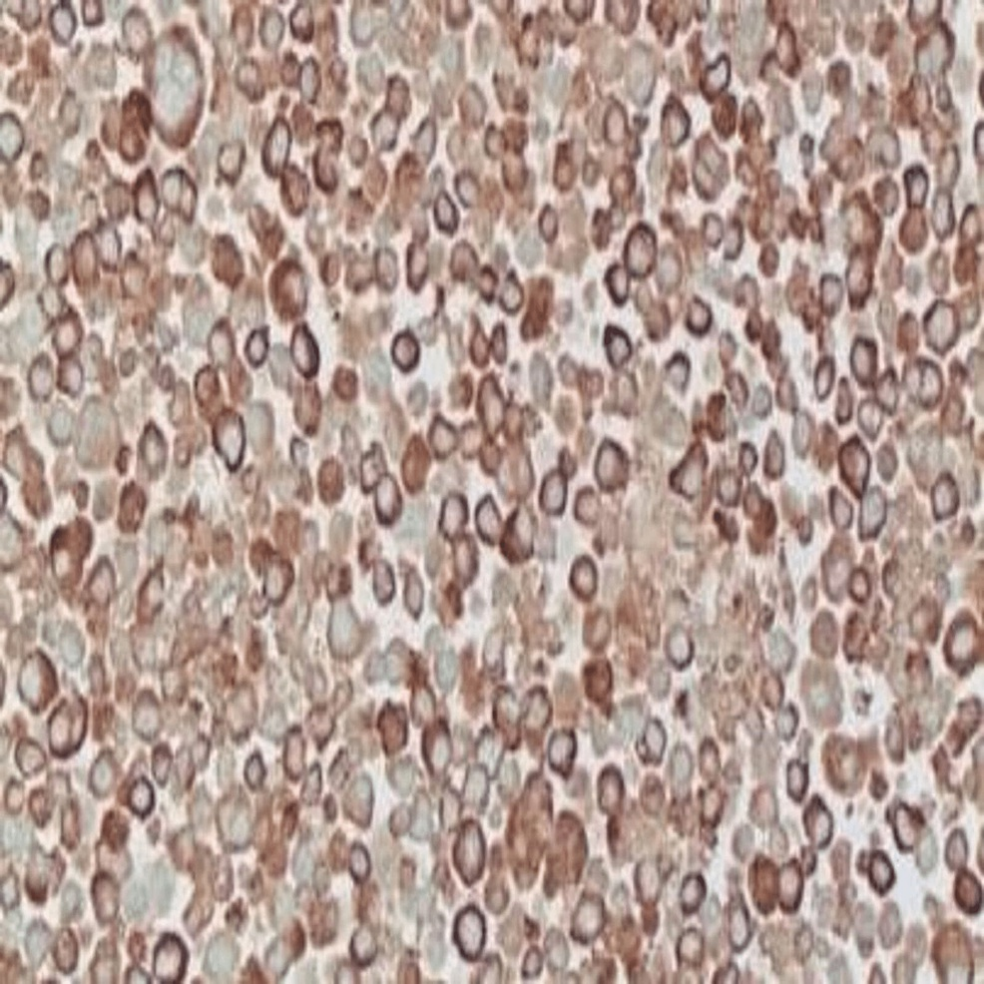
Immunohistochemistry image 2 (X 200) Plasma cells with predominant expression of lambda light chain.

**Figure 6:**
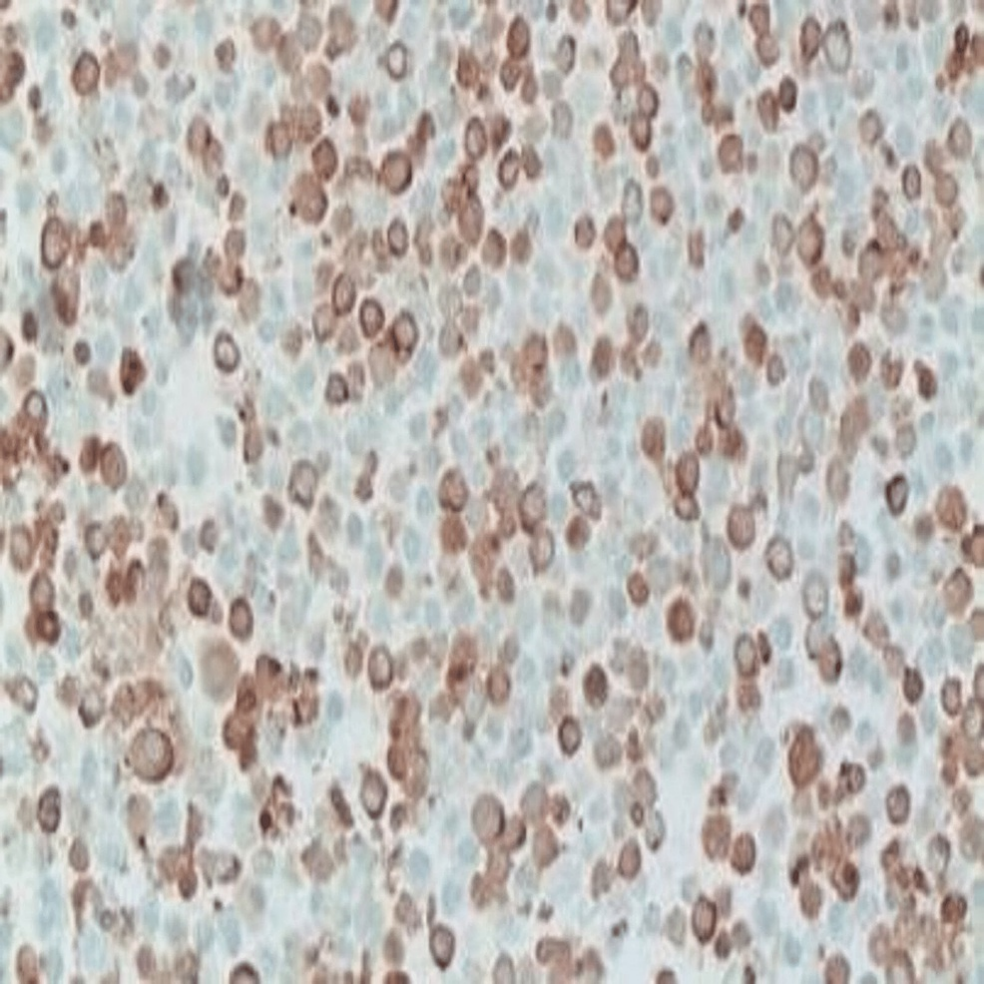
Immunohistochemistry image 3 (X 200) Plasma cell with less expression of kappa light chain.

Given the cytologic finding of atypical plasmacytoid cells, the patient was evaluated by a hemato-oncologist. He underwent a bone marrow aspiration biopsy (November 15, 2018), which revealed 2% plasma cells and less than 5% CD 138+ve cell population (negative). Flow cytometry was also unremarkable (Figures 4-6).

PET (Positron Emission Tomography) CT (Computed Tomography) scan showed 6 mm (about 0.24 in) nodules in the right lower lobe without abnormal radiopharmaceutical uptake with interval decrease in the pleural effusion; some mild pleural thickening without abnormal radiopharmaceutical uptake. His IgG, IgA, and IgM level was within normal limits. The kappa lambda ratio was normal. He was diagnosed to have solitary intramedullary plasmacytoma. There was extensive discussion of the treatment plan with the patient which included observation vs radiation therapy vs combination therapy with lenalidomide (Revlimid®) plus dexamethasone (Len/Dex). With informed decision-making, the patient wanted to proceed with close follow-up and monitoring. He was kept on active surveillance with repeat laboratory workup (CBC {complete blood count}, LDH {lactate dehydrogenase}, B2 microglobulin, SPEP (serum protein electrophoresis), immunoglobulin levels), and repeat chest X-ray (CXR) to evaluate the degree of pleural effusion. He was followed up every six months.

During the follow-up visits he did remarkably well for the next three years; he complained of progressively worsening fatigue during follow-up visits. In 2021, three years later he presented with worsening shortness of breath and gastrointestinal (GI) bleeding. On further workup, the patient was found to have severe anemia, on CXR he had right-sided pleural effusion. Repeat thoracentesis came positive for plasmacytoma. He also underwent a PET-CT scan which showed a stable 6 mm (about 0.24 inches) nodule in the right lower lobe without abnormal findings; some mild pleural thickening is seen without abnormal radiopharmaceutical uptake but showed soft tissue mass left ureter. He also was found to have worsening kidney function and left kidney hydronephrosis on a kidney scan. Fine needle aspiration of the left retroperitoneum showed findings suggestive of atypical plasmacytoid cells. Urine cytology was negative for high-grade urothelial cancer. The patient was managed medically for his gastroenterology bleed and underwent left-sided ureteral stent placement. The Patient got discharged to a rehab facility. On follow-up visits, he had progressive fatigue and limited mobility. He otherwise denied any episodes of fever, chills, nausea, or emesis. He states that he was initially doing well at the short-term rehab facility, however, because of the *Clostridium difficile* infection in the rehab he noted a significant decline in his overall health. There was discussion regarding starting the treatment for the SEMP but he wanted to continue to work with physical therapy to gain strength before he decided on any treatment options. On repeat lab work to rule out progression to MM he was found to have progression to MM.

Given the patient's multiple medical co-morbidities and worsening shortness of breath, there were concerns about progressive disease. There was extensive discussion regarding his overall frail status, treatment options, and goals of care. The plan was to consider a trial of Revlimid® and Decadron if the patient agreed. He decided to go ahead with comfort measures only after the goal of care discussion. He went home on hospice care.

## Discussion

Plasma cell neoplasm is a disorder of mature B-cells that results from an abnormal generation of cloned plasma cells. Solitary plasmacytomas can be localized originating either from bone marrow plasma cells or soft tissue plasma cells. SBP is more prevalent in older populations (50 to 80 years old) compared to young patients. Male are affected more compared to females. It is also found to affect African American populations more than other races [5]. The diagnostic criteria for solitary plasmacytoma according to Soutar et al. include the following: i) the presence of a single area of bone destruction or soft tissue mass due to clonal plasma cells with histologically normal marrow aspirate, (ii) absent or low serum/urinary level M-component, (iii) no evidence of systemic impairment due to plasma cell dyscrasia (anemia, hypercalcemia, or kidney dysfunction), or (iv) no additional lesions on a skeletal survey and magnetic resonance imaging scan of the spine [6].

The exact etiology of plasmacytoma is unclear but inhalation of chemicals, chronic stimulation, irradiation overdose, viral infection, and genetic disorders of the reticuloendothelial system could trigger it [7]. Patient symptoms can be variable depending on the involvement of the body parts. Extramedullary plasmacytoma most commonly affects head and neck regions followed by the oral cavity, tonsillar fossa, nasal cavities, and larynx. The involvement of gastroenterology systems [8], lungs and pleura are rare [9]. Patients with involvement of lung or pleura present with the complaint of shortness of breath, and hemoptysis. Differential diagnosis of plasmacytomas includes other benign and malignant conditions including pulmonary tumor, non-Hodgkin’s lymphoma, malignant melanoma vs benign lipoma, and other benign conditions.

Plasmacytoma can be diagnosed by biopsy of the mass. It can be diagnosed using routine hematoxylin and eosin stain. On histopathological examination, plasmacytoma contains the findings like enlarged, round nuclei and prominent nucleoli, and a high nucleus-to-cytoplasm ratio. The histologic picture resembles MM and contains a dense infiltrate of plasmablastic or anaplastic plasma cells that may be mature or immature [10]. The optimal treatment of solitary plasmacytoma depends upon the tumor size, invasiveness, and metastasis state, as well as systemic conditions. It can be treated with radiotherapy, surgical resection, and chemotherapy. Solitary plasmacytoma of the pleura is usually treated by either surgical resection alone or resection followed by radiation therapy. The majority of patients with solitary pulmonary plasmacytoma are treated with radiation therapy (RT) given with curative intent at a dose of 40 to 50 Gy for radical treatment over a four-week period and 10-20 Gy for palliative treatment [11,12]. Small lesions with complete resection may be cured with surgery alone and no adjuvant RT is indicated, but in tumors with incomplete resection local RT is indicated [13]. Local surgical removal of the tumor or local RT are equally efficacious. In surgical treatment, a margin of at least 2 cm (about 0.79 inches) is recommended.

The combination of treatment with surgical management and localized radiotherapy has satisfactory results with complete disease regression but in more than 50% of patients it has been reported to progress to MM within two years [14]. Those patient groups who progress to MM can benefit from the addition of novel agents, such as proteasome inhibitors (PI) or immunomodulatory imide drugs (IMID) [15]. Although novel PIs, IMIDs, and monoclonal antibodies-(MAbs) (e.g., anti-CD38 and anti-CS1/SLAMF7 monoclonal antibodies) based combination therapies, which are being routinely used for upfront and relapsed disease, have improved the survival for patients with MM in general, the outcome remains poor. The overall survival varies from 7 to 12 years from the time of diagnosis. Factors associated with unfavorable prognosis include increasing age, tumor size >5 cm, and persistent M-protein 1 year after treatment [16-18].

## Conclusions

Solitary extramedullary plasmacytoma (EMP) of pleura is an extremely rare presentation. Any patient diagnosed with EMP should be treated and followed up by an oncologist closely with surveillance for progression to MM. Nearly 50% of patients progress to MM within two years despite appropriate treatment.
